# Assessing the effects of foot strike patterns and shoe types on the control of leg length and orientation in running

**DOI:** 10.1038/s41598-024-52446-0

**Published:** 2024-01-26

**Authors:** Alessandro Garofolini, Karen J. Mickle, Patrick McLaughlin, Simon B. Taylor

**Affiliations:** 1 Institute for Health and Sport (IHES), Victoria University, Melbourne, Australia; 2 School of Environmental and Life Sciences, University of Newcastle, Ourimbah, NSW Australia

**Keywords:** Control theory, Dynamical systems

## Abstract

This research investigates the stabilization of leg length and orientation during the landing phase of running, examining the effects of different footwear and foot strike patterns. Analyzing kinematic data from twenty male long-distance runners, both rearfoot and forefoot strikers, we utilized the Uncontrolled Manifold approach to assess stability. Findings reveal that both leg length and orientation are indeed stabilized during landing, challenging the hypothesis that rearfoot strikers exhibit less variance in deviations than forefoot strikers, and that increased footwear assistance would reduce these deviations. Surprisingly, footwear with a lower minimalist index enhanced post-landing stability, suggesting that cushioning contributes to both force dissipation and leg length stability. The study indicates that both foot strike patterns are capable of effectively reducing task-relevant variance, with no inherent restriction on flexibility for rearfoot strikers. However, there is an indication of potential reliance on footwear for stability. These insights advance our understanding of the biomechanics of running, highlighting the role of footwear in stabilizing leg length and orientation, which has significant implications for running efficiency and injury prevention.

## Introduction

Running involves thousands of repetitive jump-land sequences, with each landing exerting significant force on the limb^1^. The external ground force during these landings is modulated by muscle–tendon stress and is influenced by changes in effective leg length and its stiffness^2,3^. Despite its importance, the central nervous system’s (CNS) coordination of the redundant motor elements, such as the multiple muscles and joints involved in the landing process, remains an area of intrigue. These elements are considered ‘redundant’ because there are more motor components available than are strictly necessary to perform the simple mechanical task of landing.

Leg posture during landing is a result of CNS coordination and can be represented by a kinematic vector, typically defined from the foot centre of pressure to the body centre of mass^3^. This vector's vertical and fore-aft components define effective leg length and orientation^4^, both of which are controlled by the CNS^5,6^. The influence of footwear and landing style on these control variables, particularly during the landing phase of running, is yet to be fully understood.

The uncontrolled manifold theory (UCM) by Scholz and Schöner^7^ provides a framework to study the CNS's coordination strategy during a redundant movement task^8,9^. This theory posits that the CNS exploits the redundancy in the motor system by allowing variability in motor commands that do not affect the overall task goal, thereby offering flexibility and adaptability in motor control. UCM analysis distinguishes between two crucial forms of variability in motor control: goal-irrelevant variability and goal-relevant variability. Goal-irrelevant variability represents the part of motor variability that doesn’t affect task performance, while goal-relevant variability contributes to achieving task goals. The UCM’s application to running has revealed insights into CNS coordination in novice and experienced runners^10^, and in runners under fatigue^11^, but its application to the landing phase remains unexplored.

**Figure 1:**
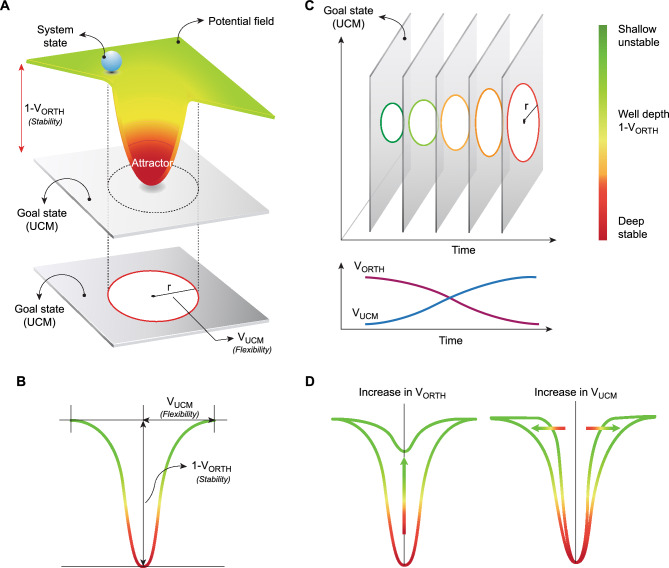
Schematic overview of variance structure of an effector system. Well analogy in 3D space, illustrating how the goal-relevant variability is dependent upon the central tendency of the system’s state where the space that the effector state is attracted upon can be expressed by the analogy of the potential energy of a well (**A**). The well’s basin width (r) represents the goal-irrelevant variability, which is the repertoire of configurations that will converge toward the goal state—i.e. flexibility. The well depth (1-V_ORTH_) represents the magnitude of goal-relevant variability, where an increased depth indicates the strength of the convergent attraction for motor solutions to meet the goal state. The depth of the well represents a resistance to system perturbations and disruptive external influences—i.e. stability (**B**). When a task evolves across time, a sequence of manifolds across the slices of time will express the design of the control system (**C**). This will be observed from the structure of variance relative to the manifold, where changes both parallel and orthogonal will describe the characteristics of the well and hence the design of the system’s controllers (**D**). This figure has been generated by researcher AG using Adobe Illustrator.

From a task performance perspective, goal-irrelevant variability represents the repertoire of body-state configurations that will converge toward the goal state (i.e. variability along the manifold space), which expresses *system flexibility*. In contrast, goal-relevant variability represents the neuromotor system’s resistance to perturbations and disruptive external influences (i.e. minimal variability distributed perpendicular to the manifold space), which expresses *system stability*. The strength of the attraction for body states to map onto the manifold space (i.e. goal state) is expressed by the analogy of the potential energy of a well (Fig. 1A). Rooted in dynamical systems theory^12^, this analogy serves as a metaphorical representation. The behavior of the system can be visualized as a ball moving in a landscape of hills and valleys, or "wells". The depth of the well symbolizes the stability of the system, indicating how strongly the system is attracted to a particular state. Conversely, the width of the well represents the flexibility of the system, showcasing the range of states the system can adopt while still achieving the task goal (Fig. 1B). This analogy, while metaphorical, offers a tangible lens to interpret the intricate behaviours of the system, bridging the gap between complex numerical data and the overarching narrative of how the locomotor system manages stability and flexibility. Assuming that the salient details of performance that map to the CNS is equivalently represented by the solution space of the UCM, then the ratio of orthogonal deviations relative to parallel deviations is evidence of a CNS plan for a motor synergy^13,14^. Here, the term *synergy* applies to the situation when the CNS achieves a motor strategy that is both flexible (well radius) and stable (well depth). As a task unfolds temporally, the evolution of the control system's design can be discerned through a time-sequenced series of manifolds (Fig. 1C). The structural organization of variance with respect to these manifolds—comprising both parallel and orthogonal fluctuations—serves as a descriptor for the control system's architecture. Specifically, variations that align with the manifold's orientation (parallel) reflect the system's adaptive strategies, while those that deviate (orthogonal) reveal the system's compensatory mechanisms. Together, these variations delineate the contours of the ‘well’ in our metaphor (Fig. 1D), providing a quantifiable measure of the control system's design parameters, namely its stability and flexibility.

The aforementioned theoretical framework sets the stage for our empirical investigation into the functional aspects of running biomechanics. Specifically, we aim to investigate the consistency of leg length and orientation during the landing phase of running and to understand how different foot landing styles and shoe types might influence this. We predict that both leg length and orientation will be controlled by the CNS, with leg length showing more consistent patterns due to the greater forces in the vertical direction. Additionally, we previously found that habitual rearfoot strike runners exhibit a more consistent pattern in controlling leg stiffness during key phases of running, implying a potentially less adaptable but more stable system^15^. This is further supported by findings from^16^, which suggest greater core stability in rearfoot strikers due to more balanced muscle activation patterns. These findings align with our hypothesis that rearfoot strikers might exhibit greater stability with less flexibility. Regarding the influence of footwear, it has been observed that shoes with more support can lead to adjustments in the overall stiffness of the leg. This adjustment is a response to the footwear's characteristics, aiming to maintain an optimal balance in leg stiffness, which is crucial for efficient running mechanics^17^. Consequently, we propose that more supportive footwear might enhance stability for rearfoot strikers by facilitating these adjustments, without necessarily increasing flexibility. Lastly, we hypothesize that forefoot strikers, particularly when wearing shoes with minimal support, will demonstrate increased flexibility, a hypothesis that requires further investigation to establish a direct comparison with rearfoot strikers in terms of adaptable control patterns.

## Methods

### Participants

Twenty male long-distance runners (age: 31.2 ± 6.9 years, height: 1.77 ± 0.07 m, weight: 73.4 ± 7.9 kg) gave their informed consent to take part in this study which was approved by the High Risk Ethics committee of Victoria University (No. HRE16-061). All research was performed in accordance with the Declaration of Helsinki. Participants were excluded if they had not been running for at least 5 years, with an average of at least 40 km/week, and had not been free of neurological, cardiovascular, or musculoskeletal problems within the previous six months. Participants were classified as rearfoot strikers (RFS, n = 10) or forefoot strikers (FFS, n = 10) based on their habitual mode of foot strike assessed by analysing the joint ankle moment from foot contact to the time of reaching 1 body weight on the vertical component of the ground reaction force. Runners who displayed a positive (dorsiflexor) moment for at least 90% of the analysed period were classified as RFS; conversely, runner who displayed a negative (plantarflexor) moment for at least 90% of the analysed period were classified as FFS. This classification is more representative of the working condition of the ankle compared to conventional methods^18^.

### Experimental protocol

Tests were performed on an instrumented treadmill at a fixed speed of 11 km/h. Kinematics of the lower extremities were recorded with a fourteen VICON camera system (Oxford Metrics Ltd, UK) at a sampling rate of 250 Hz. After a standardized 7-min progressive warm-up, participants ran for five minutes in each of the three different kinds of footwear characterised by different minimalist indexes—MI^19^ ranging from 0% (maximum structural support) to 100% (less interaction with the foot). The order of presentation was pseudo-randomized. The shoes adopted in our experiments were classified as low MI (Mizuno® Wave Rider 21, MI = 18%), medium MI (Mizuno® Wave Sonic, MI = 56%), and high MI (Vibram® Five fingers, MI = 96%). A rest period of at least 3 min was given between testing conditions.

### Data processing and analysis

**Figure 2:**
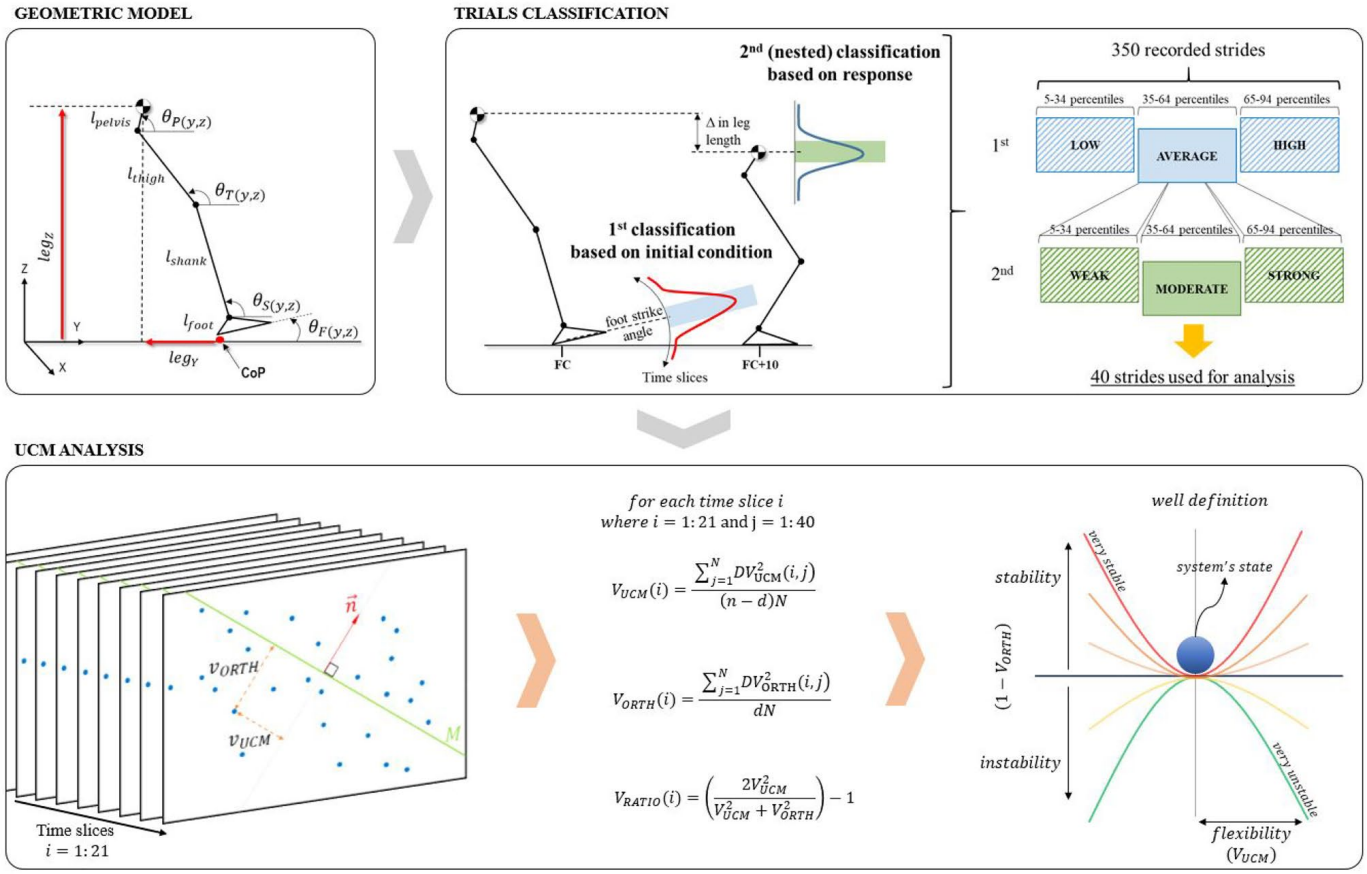
Geometric model used to estimate performance variables leg length (leg_z_) and leg orientation (leg_y_) and joint angle for pelvis (θ_P_), thigh (θ_T_), shank (θ_S_), and foot (θ_F_) segments within the sagittal plane (y,z). The leg effector is constructed from the centre of pressure (CoP) to the pelvis mid-point. Note: thigh, shank, and foot segment angles were computed on both left and right sides; the left side is not shown in the figure. Trials were ranked using foot strike as first, where the average foot strike angle (35–64 percentile) was selected; then a second (nested) classification was done by selecting the trials with a moderate (35–64 percentile) change in leg length (defined as the change during the period [FC, FC + 10]). The UCM analysis was then performed on each time slice. For each slice a linearized manifold (M) and a normal vector ($\overrightarrow{{\text{n}}}$) were computed; then the sum of all distances parallel (V_UCM_) and orthogonal (V_ORTH_) and their ratio (V_RATIO_) were calculated. Using V_UCM_ to represent flexibility and 1-V_ORTH_ to represent stability the possible well structures are shown. This figure has been generated by researcher AG using Power Point 2016.

Joint position was recorded from 21 retro-reflective markers (14 or 9 mm diameter) attached to the pelvis, thigh, shank and feet as per^20^. For the UCM analysis, the body was represented as a planar system of 7 rigid segments (pelvis, thigh × 2, shank × 2, and feet) (Fig. 2). Raw data were exported to Visual 3D (C-motion) and filtered using a low-pass Butterworth filter (4th order, zero lag) with a cut-off frequency of 15 Hz. We set this frequency based on the point where 95% of the signal's content remained intact, similar to^21^ and Sinclair, Greenhalgh^22^.

Gait events were defined using the vertical component of the ground reaction force which was low pass filtered using a 35 Hz cut-off frequency^23^. An ascending and descending threshold of 20 N identified foot contact (FC), and foot off respectively, as per Garofolini, Oppici^24^. Two other events were created 40 ms before foot contact (FC − 10), and 40 ms after foot contact (FC + 10). These events were then used to cut the period of interest and normalize the data into 21 equally distant (in time) slices, where the first 10 slices represented the pre landing phase (PRE) and last 10 slices represented the post landing phase (POST). The latter, post landing phase, can be referred to as the impact phase (≈ 50 ms)^15^. Data were then exported into Matlab (The MathWorks Inc., Massachusetts, US) to evaluate the structure of variances within the UCM framework.

### Uncontrolled manifold formulation

The UCM analysis was computed at each of the 21 slices, encompassing both the PRE and POST landing phases, as well as the FC event. Each time slice corresponded to a time period of 4 ms. According to our hypothesis, elemental variables were segment angles (i.e. θ_P_, θ_T_, θ_S_ and θ_F_), while the control variables were the vertical (Leg_Z_) and horizontal (Leg_Y_) components of the leg effector (Fig. 2). Details of the UCM formulation are reported in Supplementary material. Variance of goal-irrelevant deviations are parallel to the UCM (V_UCM_), while goal-relevant deviations are orthogonal to the UCM (V_ORTH_). A third UCM parameter V_RATIO_ was computed from the ratio of V_UCM_ and V_ORTH_^25^.

Although the UCM analysis does not require temporal order of trials, it does presume that the effector repeatedly attempts the same task-goal, from a similar initial configuration state and with similar response behaviour. In this case the task-goal was landing, configuration was the foot strike angle, and response behaviour was the change in limb length. Trials within each participant were therefore rank-ordered based on foot strike angle (three groups of trials), and then based on change in limb length (three sub-groups of trials). The mid sub-group of the average foot strike angle group (average initial conditions) was considered the most representative and therefore used for further analysis (Fig. 2).

### Visualizing system flexibility and stability: the well analogy

To visually represent the balance between flexibility and stability in our system, we employed the well analogy (Figs. 1, 2). We constructed the well by fitting a second order polynomial curve through the V_UCM_ and 1-V_ORTH_ pair of values and forcing the curve to pass through the 0,0 coordinates. Forcing the curve through the origin is consistent with the idea that at the initial state (zero perturbation), there's no deviation in the system's behaviour. In this representation, the well's width (V_UCM_) symbolizes the system's flexibility, while its depth (1-V_ORTH_) indicates either the system's stability (for positive values) or instability (for negative values). This metaphorical approach provides an intuitive way to interpret the nuanced behaviours of the system.

### Statistical analysis

Dependent variables of V_UCM_, V_ORTH_, V_RATIO_ were averaged across the 10 time-slices within both the pre-landing phase and the landing phase, and reported as group mean (group standard deviation) for each footwear condition. The vertical and fore-aft dimensions of the control variable were analysed separately. Cohen's d effect sizes for each group comparison were calculated as the difference between two means divided by the pooled standard deviation. This metric is used to assess the practical significance of the observed differences. A mixed design 3-factor (*Shoe* × *Phase* × *Group*) repeated-measures ANOVA was used to examine the interaction and main effects of within-subject factors of *Shoe* (3 levels: low MI, medium MI, high MI) and task-dependent *Phase* (2 levels: pre, post), and between-subject factor of foot loading *Group* (2 levels: forefoot, rearfoot) on the three dependent variables of V_UCM_, V_ORTH_, V_RATIO_. Tukey post-hoc analysis was used for multiple pairwise comparisons to explore all possible mean differences, given its suitability for comprehensive exploratory analysis and its ability to control the family-wise error rate effectively. A two-way repeated measures ANOVA with main factors *Group* (3 levels: rearfoot, forefoot, Zero control), and *Time* (21 levels: each time slice) was used to test whether the ratio values were statistically greater than zero (V_UCM_ > V_ORTH_), thus accepting the hypothesis of vertical and fore-aft dimension stabilisation. The Dunnet’s multiple comparison correction was applied here, given its efficiency in comparing multiple treatments against a single control condition, which in our case was the 'Zero control' group. Multiple two-way repeated measures ANOVA with between-subject factor of foot loading *Group* (2 levels: forefoot, rearfoot) and within-subject factors of *Time* (21 levels: each time slice) were used to test differences between groups within each shoe condition and leg component on dependent variables (V_UCM_, V_ORTH_, and V_RATIO_). The Sidak method was employed for correcting p values for multiple comparisons in these cases, due to its balance between error control and maintaining statistical power, especially suitable for our moderate number of comparisons. Multiple two-way repeated measures ANOVA with between-subject factor of *Shoe* (3 levels: low MI, medium MI, high MI) and within-subject factors of *Time* (5 levels: FC − 10, FC − 5, FC, FC + 5, FC + 10) were used to test differences in well characteristics between shoes within each time slice and leg component on dependent variables (V_UCM_, 1-V_ORTH_). Here, the Tukey method was again utilized for multiple comparisons, considering its appropriateness for extensive pairwise comparison in this context. Significance was set at 0.05 for all tests. All statistics were performed using GraphPad Prism (version 9.1.1, GraphPad Software, San Diego, California, USA).

## Results

**Figure 3:**
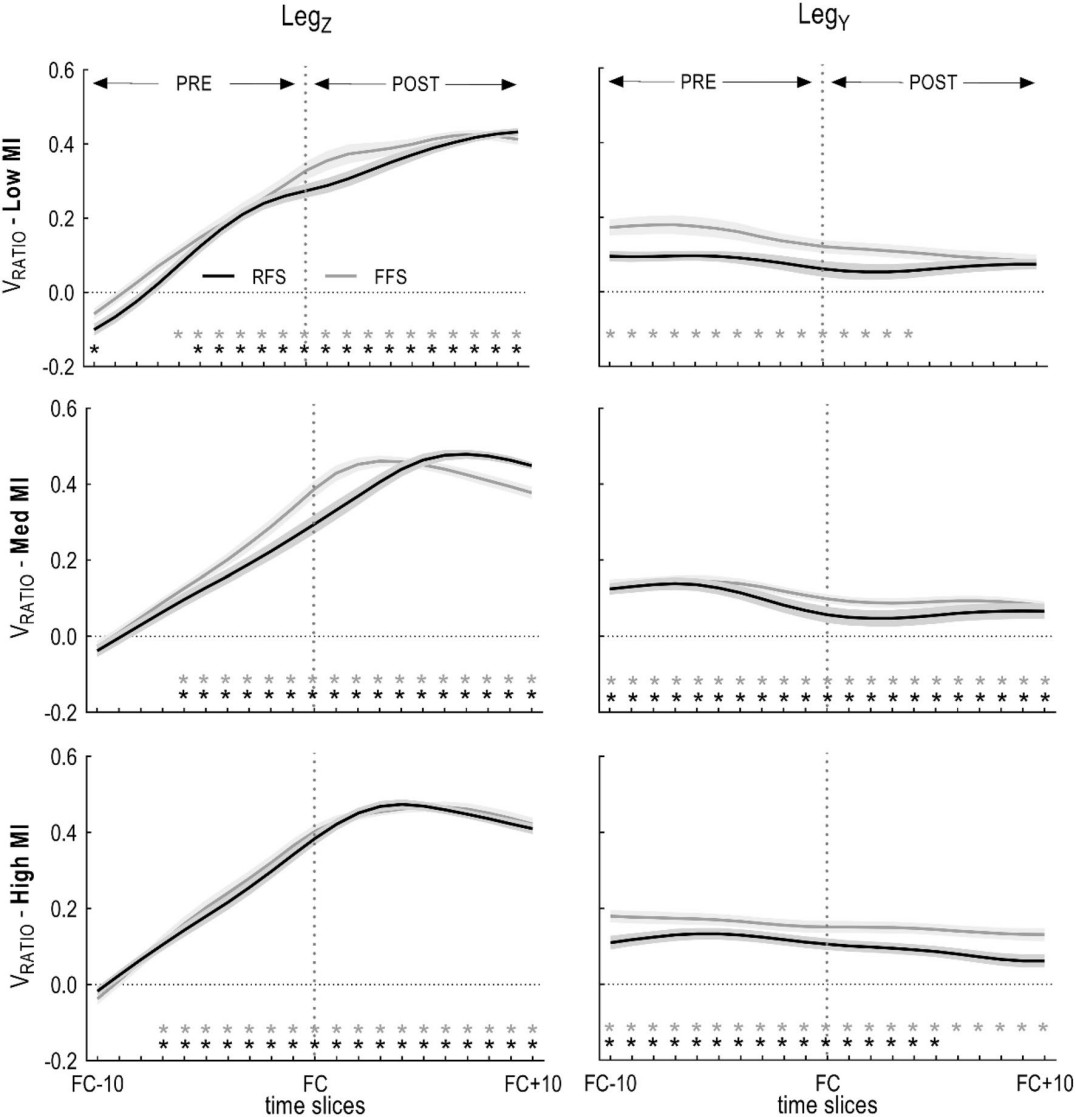
Mean ± SE ratio values for RFS and FFS groups. Time has been divided in two phases: PRE from 10 frames before foot contact (FC − 10) to foot contact (FC); and POST from FC to 10 frames after foot contact (FC + 10). Note: frames correspond to absolute time (ms); 1 frame = 4 ms. FC + 10 is ~ 15% of stance. * indicates statistically significant difference from the ‘Zero Control’ condition; grey colour refers to FFS, black colour refers to RFS group.

V_RATIO_ values for both the Leg_Z_ and Leg_Y_ control variables were statistically different from zero for most of the landing phase for all shoe conditions (Fig. 3). Although not significant, FFS displayed higher V_RATIO_ values in low MI shoes at the beginning of the PRE phase, and in high MI shoes at the end of the POST phase on the Leg_Y_ component. On the Leg_Z_ component, FFS had an earlier peak in med MI shoes than RFS.

**Figure 4:**
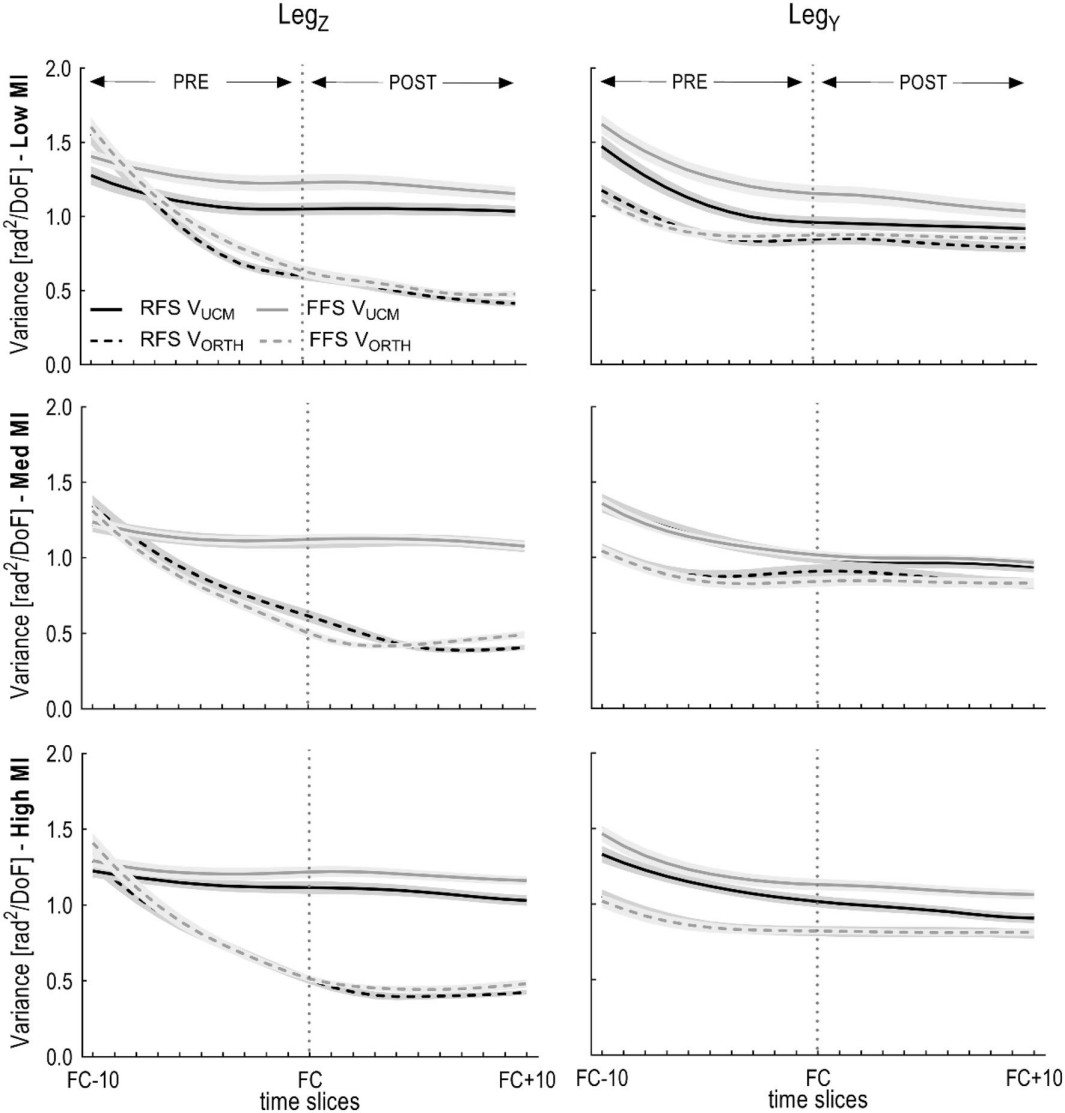
Mean ± SE of Variance components parallel (solid lines) and orthogonal (dashed lines) to the linearized UCM. Note: frames correspond to absolute time (ms); 1 frame = 4 ms. FC + 10 is ~ 15% of stance.

Figure 4 shows the V_UCM_ and V_ORTH_ along the time course of the landing phase. RFS and FFS groups show similar behaviours, V_ORTH_ decreases in the PRE phase and remains constant after foot contact, showing a statistical difference between PRE and POST phase for the Leg_Z_ (*p* < 0.001) but not for Leg_Y_ components (*p* = 0.166; Table 1). For both groups, the elbow in the curve happens earlier in low MI shoes than in med or high MI shoes. Although not significant, FFS tend to have higher values for V_UCM_ in low MI shoes in both Leg_Z_ and Leg_Y_ components.

**Table 1:** Primary statistical results for differences between Groups, Shoes, and Phase for primary variance characteristics (Table 2): variance parallel to the UCM (V_UCM_), variance orthogonal (V_ORTH_), and ratio (V_RATIO_) for the vertical component (Leg_Z_) and horizontal component (Leg_Y_).

Variable	Group	Shoe	Phase	Group × Shoe	Group × Phase	Shoe × Phase	Group × Shoe × Phase	Tukey’s Shoe effects	Phase effects
Leg_Z_									
V_UCM_	F_(1,18)_ = 0.88 *p* = .362	F_(2,36)_ = 0.33 *p* = .329	F_(1,18)_ = 1.33 *p* = .264	F_(2,36)_ = 1.47 *p* = .244	F_(1,18)_ = 0.15 *p* = .705	F_(2,36)_ = 0.28 *p* = .761	F_(2,36)_ = 0.22 *p* = .802		
V_ORTH_	F_(1,18)_ = 0.13 *p* = .723	**F**_**(2,36)**_** = 6.36 *p* = .007**	**F**_**(1,18)**_** = 104.6 *p* < .001**	F_(2,36)_ = 3.335 *p* = .079	F_(1,18)_ = 0.02 *p* = .892	F_(2,36)_ = 0.53 *p* = .592	F_(2,36)_ = 1.36 *p* = .270	**Low > med: *p* = .003** **Low > high: *p* = .003** Med > high: *p* = .739	**Pre > Post** ***p* < .001**
V_RATIO_	F_(1,18)_ = 0.43 *p* = .518	**F**_**(2,36)**_** = 7.95 *p* = .006**	**F**_**(1,18)**_** = 221.8 *p* < .001**	F_(2,36)_ = 0.47 *p* = .534	F_(1,18)_ = 0.11 *p* = .743	F_(2,36)_ = 0.37 *p* = .692	F_(2,36)_ = 0.41 *p* = .666	**Low < med: *p* = .043** **Low < high: *p* < .001** Med < high: *p* = .063	**Pre < Post** ***p* < .001**
Leg_Y_									
V_UCM_	F_(1,18)_ = 1.19 *p* = .290	F_(2,36)_ = 0.74 *p* = .432	**F**_**(1,18)**_** = 5.79 *p* = .027**	F_(2,36)_ = 1.98 *p* = .173	F_(1,18)_ = 0.17 *p* = .689	F_(2,36)_ = 0.26 *p* = .772	F_(2,36)_ = 0.22 *p* = .803		**Pre > Post** ***p* = .027**
V_ORTH_	F_(1,18)_ = 0.02 *p* = .896	F_(2,36)_ = 0.89 *p* = .409	F_(1,18)_ = 2.08 *p* = .166	F_(2,36)_ = 0.38 *p* = .623	F_(1,18)_ = 0.06 *p* = .808	F_(2,36)_ = 0.29 *p* = .750	F_(2,36)_ = 0.12 *p* = .890		
V_RATIO_	F_(1,18)_ = 4.39 *p* = .051	F_(2,36)_ = 1.29 *p* = .287	F_(1,18)_ = 3.83 *p* = .066	F_(2,36)_ = 0.28 *p* = .723	F_(1,18)_ = 0.004 *p* = .949	F_(2,36)_ = 0.09 *p* = .909	F_(2,36)_ = 0.23 *p* = .799		

ANOVA results are given for main effects and interactions. Given that interactions effects were not significant, the Tukey’s pairwise comparisons results are reported only for the main effect of shoe. Low = low minimal index shoe; med = medium minimal index shoe; high = high minimal index shoe. Statistically significant findings are in bold.

### Variance parallel to the UCM, V_UCM_

There was a main effect of *Phase* (*p* = 0.027; Table 1) only for the horizontal (Leg_Y_) control variable. Indicating that on average V_UCM_ was dependent on the phase of the landing task only in Leg_Y_. Post-hoc analysis revealed that V_UCM_ pre landing is higher compared to post landing for Leg_Y_; while it stays statistically unchanged for Leg_Z_ (Fig. 4, Table 2).

**Table 2:** Mean ± standard deviation and Cohen's d effect sizes for variance parallel (V_UCM_), orthogonal (V_ORTH_), and ratio (V_RATIO_) across the three footwear conditions for the vertical (Z) component and horizontal (Y) component.

Z component	PRE			POST		
	RFS	FFS	Cohen’s d	RFS	FFS	Cohen’s d
Low MI						
V_UCM_	1.12 ± 0.31	1.28 ± 0.30	− 0.52	1.03 ± 0.30	1.09 ± 0.44	− 0.15
V_ORTH_	0.98 ± 0.25	1.05 ± 0.26	− 0.27	0.49 ± 0.14	0.52 ± 0.13	− 0.21
V_RATIO_	0.09 ± 0.10	0.12 ± 0.11	− 0.27	0.37 ± 0.08	0.40 ± 0.08	− 0.38
Med MI						
V_UCM_	1.14 ± 0.29	1.15 ± 0.19	− 0.04	1.08 ± 0.30	1.05 ± 0.28	0.11
V_ORTH_	0.95 ± 0.26	0.88 ± 0.15	0.29	0.44 ± 0.12	0.45 ± 0.10	− 0.09
V_RATIO_	0.11 ± 0.11	0.15 ± 0.08	− 0.36	0.44 ± 0.09	0.43 ± 0.06	0.13
High MI						
V_UCM_	1.16 ± 0.23	1.23 ± 0.26	− 0.27	1.05 ± 0.30	1.12 ± 0.30	− 0.23
V_ORTH_	0.87 ± 0.24	0.91 ± 0.24	− 0.17	0.41 ± 0.10	0.46 ± 0.12	− 0.42
V_RATIO_	0.16 ± 0.10	0.17 ± 0.10	− 0.10	0.45 ± 0.06	0.45 ± 0.07	0.00

Y component	PRE			POST		
	RFS	FFS		RFS	FFS	
Low MI						
V_UCM_	1.15 ± 0.33	1.33 ± 0.36	− 0.51	0.90 ± 0.33	0.97 ± 0.48	− 0.16
V_ORTH_	0.94 ± 0.17	0.93 ± 0.16	0.06	0.82 ± 0.21	0.87 ± 0.10	− 0.26
V_RATIO_	0.09 ± 0.09	0.16 ± 0.14	− 0.53	0.06 ± 0.11	0.10 ± 0.11	− 0.36
Med MI						
V_UCM_	1.16 ± 0.29	1.15 ± 0.17	0.04	0.93 ± 0.30	0.90 ± 0.32	0.09
V_ORTH_	0.92 ± 0.24	0.89 ± 0.21	0.13	0.87 ± 0.25	0.84 ± 0.21	0.13
V_RATIO_	0.12 ± 0.10	0.13 ± 0.09	− 0.10	0.06 ± 0.14	0.09 ± 0.07	− 0.23
High MI						
V_UCM_	1.15 ± 0.25	1.25 ± 0.27	− 0.37	0.91 ± 0.33	0.99 ± 0.36	− 0.22
V_ORTH_	0.90 ± 0.20	0.88 ± 0.20	0.10	0.81 ± 0.20	0.82 ± 0.12	− 0.06
V_RATIO_	0.12 ± 0.09	0.17 ± 0.09	− 0.56	0.08 ± 0.09	0.14 ± 0.09	− 0.67

The effect sizes were calculated to provide a measure of the magnitude of differences between groups.

### Variance orthogonal to the UCM, V_ORTH_

Inverse results were found when testing the differences in variance orthogonal to the UCM (Table 1). There was a main effect of *Phase* (*p* < 0.001) for the vertical (Leg_Z_) control variable. Post-hoc analysis revealed that V_ORTH_ pre landing is higher compared to post landing for Leg_Z_; while it stays statistically unchanged for Leg_Y_*. Shoe* had a significant main effect on V_ORTH_ (*p* = 0.007) for the vertical component only. Post-hoc tests reveal that V_ORTH_ is higher in low MI shoes compared to med MI and high MI (*p* = 0.003; *p* = 0.003, respectively), indicating that more supportive shoes may induce V_ORTH_ to increase (Fig. 4, Table 2).

### Ratio of variances perpendicular and orthogonal to the UCM, V_RATIO_

There was a main effect of *Phase* (*p* < 0.001, Table 1) for the vertical (Leg_Z_) control variable. Post-hoc analysis revealed that V_RATIO_ pre landing is lower compared to post landing for Leg_Z_; while it stays statistically unchanged for Leg_Y_*. Shoe* had a significant main effect on V_RATIO_ (*p* = 0.006) for the vertical component only. Post-hoc tests reveal that V_RATIO_ is higher in high MI shoes compared to low MI shoes (*p* = 0.063), and med MI shoes compared to low MI shoes (*p* = 0.043) indicating that more supportive shoes may induce V_RATIO_ to decrease (Fig. 3, Table 2).

### Flexibility and stability

**Figure 5:**
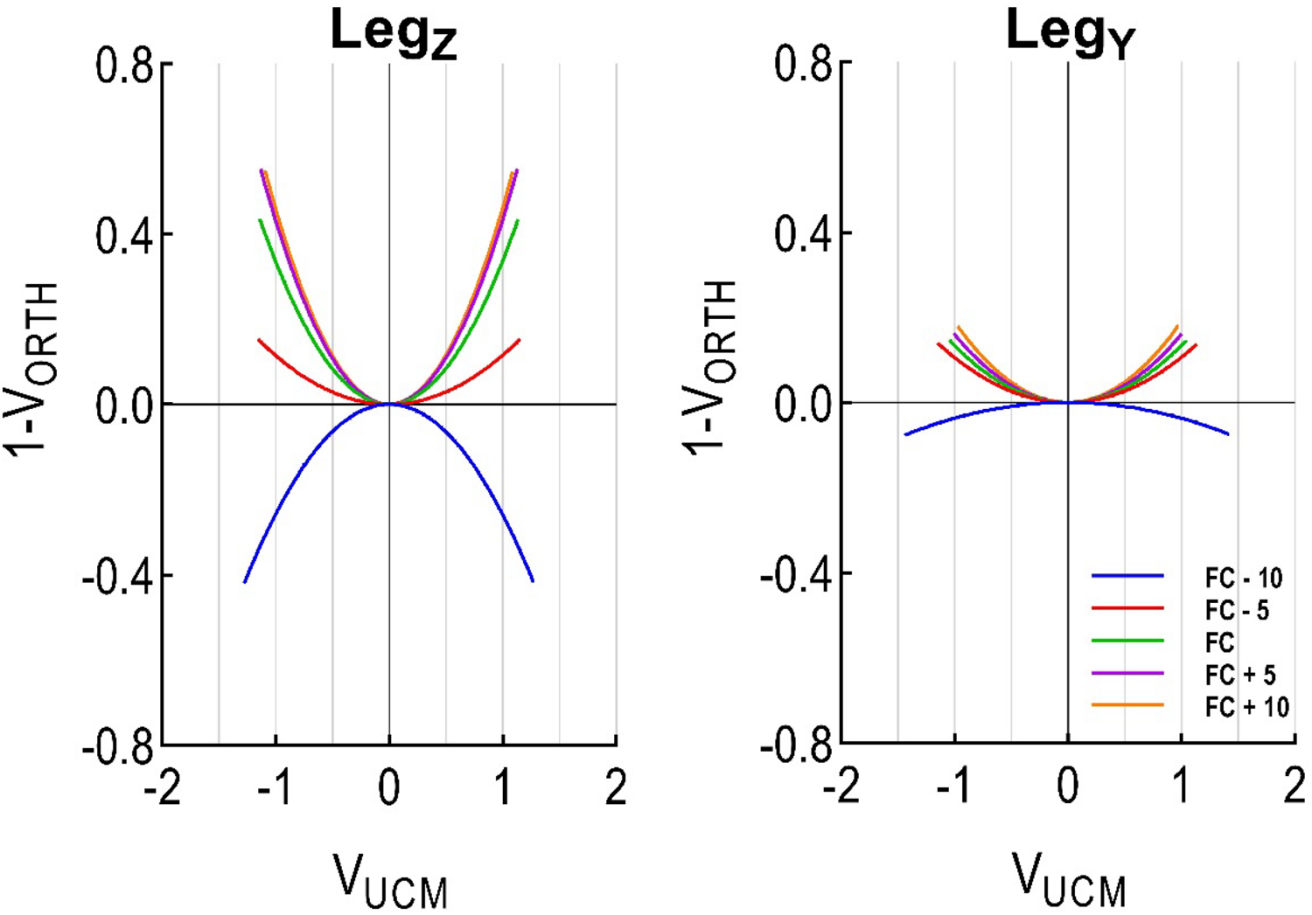
Stability (1-V_ORTH_) and flexibility (V_UCM_) using the well analogy. Groups and shoes have been merged. Five time slices where compared: Foot Contact (FC), ten time slices before foot contact (FC − 10), five time slices before foot contact (FC − 5), 5 time slices after foot contact (FC + 5), ten time slices after foot contact (FC + 10).

Given there were no statistical differences between groups in the two control variables (Leg_Z_, Leg_Y_), we merged the results from RFS and FFS (Fig. 5, Table 3) to analyse flexibility and stability using the well analogy for state attraction to the task manifold (Fig. 1). There was a main effect of *Time* (*p* < 0.0001) for both the vertical (Leg_Z_) and horizontal (Leg_Y_) control variables. Post-hoc analysis revealed the following (Table 3): (i) at FC − 10 (~ 40 ms pre foot contact) both control variables are in an unstable and highly flexible state; and (ii) at FC − 5 (~ 20 ms pre foot contact) there is a significant change for both control variables (*p* < 0.0001), where their states can be considered significantly more stable but less flexible. Leg_Z_ control variable reaches highest stability at post landing, with values 3 times higher than Leg_Y_. For both control variables flexibility decreases as a function of time (the well basin narrows as limb loading evolves).

**Table 3:** Post-hoc analysis comparing each time slices for flexibility (VUCM) and stability (VORTH) within each control variable (Leg_Z_ and Leg_Z_).

Tukey’s multiple comparisons test	Leg_Z_		Leg_Z_	
	V_UCM_	1-V_ORTH_	V_UCM_	1-V_ORTH_
FC − 10 vs. FC − 5	0.12	− 0.57**	0.29**	− 0.22**
FC − 10 vs. FC	0.14*	− 0.86**	0.39**	− 0.22**
FC − 10 vs. FC + 5	0.15*	− 0.97**	0.43**	− 0.24**
FC − 10 vs. FC + 10	0.19**	− 0.97**	0.46**	− 0.26**
FC − 5 vs. FC	0.01	− 0.29**	0.10	− 0.01
FC − 5 vs. FC + 5	0.02	− 0.40**	0.14	− 0.02
FC − 5 vs. FC + 10	0.07	− 0.40**	0.18**	− 0.04
FC vs. FC + 5	0.01	− 0.11	0.04	− 0.01
FC vs. FC + 10	0.05	− 0.11	0.07	− 0.03
FC + 5 vs. FC + 10	0.04	0.00	0.04	− 0.02

*Indicates significant differences at *p* < 0.05, **Indicates significant differences at *p* < 0.001.

## Discussion

In this study we sought to determine whether leg length and orientation are stabilized during the landing phase of running; and whether foot strike pattern and footwear affect such stability. To this aim, we asked two types of runners, based on their habitual foot strike posture at landing, to run in different footwear on an instrumented treadmill. We had three main hypotheses. (1) Both leg length and leg orientation will be stabilized during the landing phase; this was confirmed from the presence of a decrease in variance of goal-relevant deviations (i.e. V_ORTH_). (2) RFS will have comparatively less variance of both goal-irrelevant deviations (V_UCM_) and goal-relevant deviations (V_ORTH_); this was not supported. (3) Increased footwear assistance would reduce the variance of goal-relevant deviations more than goal-irrelevant deviations for RFS, this was also not fully supported by the results.

### Kinematic synergy in landing: leg length and orientation

Control variables leg length and orientation are stabilized through a kinematic synergy during landing, irrespective of landing type. The V_RATIO_ was significantly greater than zero (Fig. 3, Table 1), and a rapid reduction in V_ORTH_ occurred as the pre-landing phase evolved, demonstrating that control over limb length and orientation is a relevant goal of the locomotor control system. In contrast, goal-irrelevant variance (V_UCM_) remained relatively constant, and remarkably larger than V_ORTH_ (i.e. V_UCM_ > V_ORTH_), meaning that variance is structured to provide increased stability of leg length and orientation as the impact phase approached (V_RATIO-POST_ > V_RATIO-PRE_). In the well analogy of stability (Fig. 1), this means that the depth of the well increases while its width remains relatively unchanged (Fig. 5). These trends are indications of a locomotor control system adhering to a minimal intervention principle^26^. Our results support the idea that stabilisation of control variables (leg length and orientation) is under a hierarchical control system and subjected to a higher-level cost policy that is task-relevant. The idea of a strong synergy being responsible for stability of the kinematic leg effector during late swing and early stance of running is in agreement with previous studies^5,27–29^. The general findings from Ivanenko, Cappellini^5^ (method of segment angle covariance) indicate that kinematic properties of leg length and orientation are important global parameters encoded within the CNS. The general concept of synergy is consistent, although the particular approach taken to this conclusion of synergy is different. The segment angle covariance from Ivanenko’s principal component analysis (PCA) shows two major components. If one synergy (PCA component 1) relates to limb orientation, while the other synergy (component 2) relates to limb length, then each synergy should be evident from the UCM analysis if variance anisotropy relative to a solution manifold is revealed. While the PCA method was used to identify a synergy (i.e. limb axis length and orientation), the UCM in our study quantifies the hypothesized synergy somewhat more precisely (i.e. flexibility and stability of the synergy).

### Differential stabilization: leg length vs. leg orientation

We found that both control variables demonstrated relatively similar V_UCM_, however, they differed in the way that V_ORTH_ was reduced (Fig. 4, Table 2). The leg length (Leg_Z_) was stabilized rapidly prior to foot contact, indicated by the rapid decrease in V_ORTH_. This rapid reduction in V_ORTH_ may represent the convergence toward a stable passive attractor (a deep well in Figs. 1, 5) where higher level intervention is not necessary to provide task stability (passive attractor). The tools to appropriately reconcile the responsible source of this rapid change in V_ORTH_ would require extended analysis that combines surrogate data sets where the segment angle correlations are randomised to reduce their non-trivial covariance structure and then perform the UCM method^30^. Nevertheless, we can reasonably conclude that a consistent leg length is a goal for the landing task of running. In contrast, the leg orientation (Leg_Y_) was relatively less stable during the same period, indicated by a modest reduction in V_ORTH_. However, the V_RATIO_ of leg orientation was significantly greater than 0, indicating that there is a significant non-trivial structuring of the covariance – *a synergy*. Control of leg orientation (i.e. fore-aft dimension) has implications with braking forces and it has different implications for RFS and FFS^31^. A RFS enables forwards migration of the centre of pressure, while a FFS opposes this motion. The consequence of a restricted forwards migration leads to an increase in the braking impulse^32^.

### Influence of footwear on stability and flexibility

Leg posture (length and orientation) at landing determines stance goals, such as efficiency, stable trajectory of body centre of mass^33^, and loading stresses^34^. There is no direct evidence in the literature to indicate which dimension of leg posture contributes most to these goals; however, because Leg_Z_ stability continues to increase as landing evolves (Figs. 4, 5), we argue that Leg_Z_ is relatively more important than Leg_Y_ to meeting these essential goals of stance. Indeed, control of both leg length and orientation is important; however, a kinematic effector system cannot simultaneously optimize orthogonal dimensions when each dimension shares common elemental properties. From our results it appears that the locomotor control system is designed to prioritise the vertical dimension. By stabilizing leg length pre landing, the locomotor control system sets consistent initial conditions to control leg stiffness post landing^17,35–38^. The transient foot–ground impact forces are easily perturbed, and the control system relies less upon feedback-driven corrections to the limb’s dynamic state^39^. Therefore, stability of leg length pre-landing is likely a pre-emptive control strategy to in-turn control the associated properties of leg stiffness and musculoskeletal forces upon landing. It is worth noticing that stability of leg length post landing seems affected by the shoe substrate (Fig. 4): low MI shoes provide more stability post landing (descending values of V_ORTH_) compared to the Med MI and high MI shoes where V_ORTH_ reaches a minimum and stays constant afterwards, indicating that cushioning may not only help with dissipation of external forces^40^ but also with stability of leg length. Our results offer a new insight in understanding running related injuries: shoe type seems to negatively affect the kinematic stability of the leg effector system, and while the associated effects are unknown, it is likely that a lack of leg length stability will have a negative effect on tissue load and energy efficiency. This instability can be particularly evident in real-world running scenarios, such as when a runner encounters an unexpected increase in ground height, like a rock, curb, or slope. In such situations, if the leg is not in the desired position to accept body weight due to altered stability, it can lead to stumbling or increased difficulty in maintaining efficient running mechanics.

### Rearfoot landing: implications for flexibility and shoe assistance

The hypothesis that habitual rearfoot landing restricts the flexibility of the two control variables was not statistically supported (Fig. 4, Table 2), although the results indicate a trend from two footwear conditions (low MI and high MI shoes) where RFS showed a lower V_UCM_. Any decrease of the observed V_UCM_ may represent a reduction in the neuromotor repertoire of flexible solutions, and evidence of a system designed for minimal intervention that can default to the low-level allometric control process^41^. Possessing an abundant and flexible system could be important if the landing phase requires a rapid movement solution from low-level control mechanisms^42,43^. The RFS group may have restricted their available degrees of freedom and the potential to find flexible motor solutions for landing, and in consequence they may become dependent upon the shoe for assistance^44^. The implications for a RFS runner relate to situations when they are faced with alternate conditions. For example, many athletes habituate to a RFS technique but may perform in endurance competitions where they use high MI footwear. Future research will need to determine if RFS-related loss in landing flexibility exists and persists, leading to loss of energy efficiency and increased injury risk when faced with situations that impose alternate substrate conditions. Additionally, it would be valuable to explore what happens when habitual rearfoot strikers are presented with scenarios requiring a more adaptable control strategy, such as a change in foot striking pattern in response to certain perturbations or testing constraints. In these situations, the enhanced stability or reduced number of solutions typically experienced by rearfoot strikers could potentially lead to decreased energy efficiency^45,46^ and an increased injury risk^47^. This consideration is particularly relevant in understanding how habitual movement patterns adapt to unexpected changes in running dynamics.

### Study limitations and assumptions

A limitation of this study is related to the biomechanical model, as we assumed the central point of the pelvis to be the goal position of the effector endpoint, although the true goal of the effector endpoint is likely to be the body CoM. When interpreting the results of the UCM method we are unable to distinguish which joint is more responsible for effector endpoint change. Specifically, we did not test the sensitivity of the effector endpoint position to individual joints as we assume the effector endpoint position will change an equivalent displacement per change in unit radian of each elemental variable. It may be that the system becomes more sensitive to certain elemental variables in unfamiliar conditions^48^. Another limitation of the study is the limited sample size. To address this, in addition to standard statistical analyses, we computed Cohen's d effect sizes for each comparison (Table 2). Reporting effect sizes allows us to provide a nuanced understanding of the data, complementing inferential statistics and offering insights into the potential real-world implications of our findings.

### Conclusion

In conclusion, FFS and RFS are equally able to reduce task-relevant variance in order to stabilize the control variables leg length and leg orientation. The assistance provided by the shoe may affect the control variables (leg length and orientation) with possible implications for force control at landing.

### Supplementary Information

Supplementary Information.

## Data Availability

The datasets generated during and analysed during the current study are available from the corresponding author on reasonable request.
