# Supplementary material for: Assessing the effects of foot strike patterns and shoe types on the control of leg length and orientation in running
Source: Sci Rep. 2024 Jan 26;14:2220. doi: 10.1038/s41598-024-52446-0 (PMC10817954; doi:10.1038/s41598-024-52446-0)
Supplement: Supplementary file 1 — Supplementary Information. [file 41598_2024_52446_MOESM1_ESM.docx]

## ****Assessing the Effects of Foot Strike Patterns and Shoe Types on the control of Leg Length and Orientation in Running****

### **Supplementary Material**

### **Uncontrolled Manifold formulation**

To understand how the locomotor control is addressing the problem of ubiquitous variance in the redundant kinematic leg effector system (segment angles) are being coordinated and controlled during the leg landing phase of running, to achieve consistency in the control variables of the 2D leg effector end-point (i.e. the leg orientation and length, *Leg_Y_, Leg_Z_* respectively). The set of covariant solutions of these elemental variables (i.e. foot, shank, thigh, and pelvis angles, $\theta_{F},\theta_{S}, \theta_{T},\theta_{P}$ respectively) is defined as a low-dimensional synergy that work together in order to assist the controller by stabilizing (or destabilize) the control variable. We consider the kinematic leg effector vector (spanning the kinematic segment chain) is coordinated and controlled by a two-level motor control hierarchy [1, 2].

The UCM analysis was computed at each time slice of the landing phase period. Each time slice corresponded to a time period of 4ms. The UCM parameters were calculated using a customised Matlab program that was based on the conventional UCM method [2]. The UCM analysis method can be described in four general steps below:

#### **Step 1: Define the Geometric Model**

The kinematic leg effector is defined by a geometric function that maps the segment angles with the 2D effector end-point:

|  | $\left( L_{Y},L_{Z} \right)= f(\theta_{F},\theta_{S}, \theta_{T},\theta_{P})$ | (1) |
| --- | --- | --- |

The leg effector was defined by a vector spanning between a fixed point at the centre of the pelvis segment and the location of the centre of pressure beneath the foot. From equation 1 the 2D position of the leg effector is defined by a specific geometric model that directly maps the end-effector in the same space as the elemental variables. The control variables of *Leg_Y_* and *Leg_Z_* are associated geometrically with the elemental variable details, segment angles and segment lengths:

|  | $\left\{ \begin{aligned} \boldsymbol{L}_{\boldsymbol{Y}}= l_{ft}\cdot\cos\left( \theta_{F} \right)+l_{sh}\cdot\cos\theta_{S}+l_{th}\cdot\cos\theta_{T}+l_{pv}\cdot\cos\theta_{P} \\ \boldsymbol{L}_{\boldsymbol{Z}}= l_{ft}\cdot\sin\left( \theta_{F} \right)+l_{sh}\cdot\sin\theta_{S}+l_{th}\cdot\sin\theta_{T}+l_{pv}\cdot\sin\theta_{P} \end{aligned} \right.$ | (2) |
| --- | --- | --- |

where *l_ft_* , *l_sh_* , *l_th_* , and *l_pv_* are the lengths of the foot, shank, thigh, and pelvis respectively; while $\theta_{F}$, $\theta_{S}$, $\theta_{T}$, and $\theta_{P}$ are the segment angles (with respect to the horizontal axes).

#### **Step 2: Linear Approximation of the UCM**

A deviation matrix (***DV***) from the mean joint configuration at each *i*th time instant was computed for each *j*th stride:

|  | ${\boldsymbol{DV}(i,j)}= \left[ \begin{matrix} \theta_{F}\left( i,j \right)-\overline{\theta}_{F}(i) \\ \theta_{S}\left( i,j \right)-\overline{\theta}_{S}(i) \\ \theta_{T}\left( i,j \right)-\overline{\theta}_{T}(i) \\ \theta_{P}\left( i,j \right)-\overline{\theta}_{P}(i) \end{matrix} \right]$ | (3) |
| --- | --- | --- |
|  |  |  |

The Jacobian matrix (***J***) relating partial changes in elemental variables (i.e. $\theta_{F},\theta_{S}, \theta_{T},\theta_{P}$) to partial changes in the control variables (i.e. ${Leg}_{Y},{Leg}_{Z}$), was computed around the mean joint configuration (i.e. $\overline{\theta}_{F},\overline{\theta}_{S}, \overline{\theta}_{T},\overline{\theta}_{P}$) across the set of strides (trials) for each time slice of the period (*i*=21).

#### Step 3: Projecting the joint configuration **onto the UCM**

The next step was to compute the null space of the Jacobian matrix (***N(J)***). The null space is the linear subspace of all the segment angle combinations that result in no change to the end-effector position [3]. The null space spanned by the basis vectors $\varepsilon_{n-d}$ has a dimension equal to the difference between the number of elemental variables (*n=4*) and the number of control variables (*d=2*).

|  | ${\boldsymbol{N}(\boldsymbol{J})}= \left[ \begin{matrix} \varepsilon_{11} & \varepsilon_{12} \\ \varepsilon_{21} & \varepsilon_{22} \\ \varepsilon_{31} & \varepsilon_{32} \\ \varepsilon_{41} & \varepsilon_{42} \end{matrix} \right]$ | (4) |
| --- | --- | --- |
|  |  |  |

The deviation matrix then decomposed into components parallel (${DV}_{\parallel}$) and perpendicular (${DV}_{\perp}$) to the null space:

|  | ${DV}_{\parallel}(i,j)=\sum_{k=1}^{n-d} \left( {\mathbf{N}\left( \mathbf{J} \right)}_{i}^{T} \cdot\boldsymbol{DV}\left( i,j \right) \right)\cdot{\boldsymbol{N}(\boldsymbol{J})}_{k}$ | (5) |
| --- | --- | --- |
|  | ${DV}_{\perp}\left( i,j \right)=\boldsymbol{DV}\left( i,j \right)-{DV}_{\parallel}(i,j)$ | (6) |

#### **Step 4: Computing the variance of V_UCM_ and V_ORTH_**

The variance of these projections were estimated and normalized per degree of freedom of each subspace as:

|  | $\sigma_{\parallel}^{2}(i)=\frac{\sum_{j=1}^{N} {DV}_{\parallel}^{2}(i,j)}{\left( n-d \right)N}$ | (7) |
| --- | --- | --- |
|  | $\sigma_{\perp}^{2}(i)=\frac{\sum_{j=1}^{N} {DV}_{\perp}^{2}(i,j)}{dN}$ | (8) |

Variance of goal-irrelevant deviations are parallel to the UCM are indicated as V_UCM_ ($\sigma_{\parallel}^{2}(i)$) , and goal-relevant deviations are orthogonal to the UCM are indicated as V_ORTH_ ($\sigma_{\perp}^{2}\left( i \right)$). The variances were computed at each time instance and compared across conditions. The ratio of variability V_RATIO_ was the third UCM parameter and was computed in a form suggested by Papi, Rowe [4]:

|  | $Ratio=\left( \frac{{2\sigma}_{\parallel}^{2}}{\sigma_{\parallel}^{2}+\sigma_{\perp}^{2}} \right)-1$ | (9) |
| --- | --- | --- |

this formulation expresses the ratio in a range from -1 and +1 with 0 as midpoint avoiding the symmetrical and statistical problem related to the original formulation $\sigma_{\parallel}^{2}$/$\sigma_{\perp}^{2}$ [5]. This ratio reflects the need for intervention in order to control the control variable during landing. If the ratio is greater than 0, the effector system has a coordination strategy that produces a stable goal variable and is indicative of motor redundancy. On the contrary, ratios less than 0 will indicate that variations in coordination will have a larger effect on the control variable.

***Comparison of main covariance-based methods***

Among many covariance-based methods (e.g. Principal Component Analysis), the UCM has an advantage because it uses a geometric function that maps the null space of covariant local body-state variables (e.g. segment angles) to a geometric manifold that represents the equivalent set of solutions that satisfies a global control variable (e.g. effector endpoint position) [6]. From repeated trials of the task, the observed variability that lies unequally distributed relative to orthogonal dimensions of the manifold (UCM) is an expression of neuromotor organisation by the CNS, which seeks a strategy to achieve stable and flexible goal state solutions with minimal intervention [7].

The principal component analysis - PCA method [8] describes how body state variables form collective components without any priori knowledge of geometric relatedness to task goal. This makes PCA methods difficult to interpret. The UCM, goal equivalent manifold - GEM and tolerance noise and covariance - TNC methods are all covariance-based analysis that map body state variables to a task goal (solution manifold). The GEM method [9] is conceptually similar to the UCM, except it provides additional information about the error surrounding the task goal. Both GEM and UCM methods can be applied to multiple body state variables [10, 11]. The exception is that the GEM method provides additional information about the performance error, which can be beneficial if known a priori that an explicit task goal exists.

The TNC method [12] differs slightly from both UCM and GEM because it requires a low dimensional mapping function (confined to 2D) that relates body state variables (i.e. position and velocity) with result/goal variables [13]. The TNC method also requires the design of a task that has an explicit goal to determine the performance error. The TNC method creates a solution manifold in 2D space, where the third dimension defines performance error (conceptually similar to GEM).

The UCM method has an advantage over PCA because UCM quantifies goal-relevant variance structure, which provides more meaningful insight into the neuromuscular system and control variables (anisotropy of variance along a solution manifold).

The UCM method has an advantage over the TNC method because it can map multiple body state variables to a task goal.

While the UCM and GEM methods are similar, the UCM satisfies the objectives of our investigation in the following way. First, our participant runners do not have knowledge of results about a target end-effector position during the impact phase, so we cannot quantify error. Second, the leg effector length and its stiffness are redundant in their contribution to external force, and we don’t know which of these three variables is the goal variable. The UCM analysis will however, show evidence that the CNS controls leg length and orientation, potentially in relation to stiffness.

**References**

1. Diedrichsen, J., R. Shadmehr, and R.B. Ivry, *The coordination of movement: optimal feedback control and beyond.* Trends in cognitive sciences, 2010. **14**(1): p. 31-39.

2. Scholz, J.P. and G. Schöner, *The uncontrolled manifold concept: identifying control variables for a functional task.* Experimental brain research, 1999. **126**: p. 289-306.

3. Latash, M.L., J.P. Scholz, and G. Schoner, *Toward a new theory of motor synergies.* MOTOR CONTROL-CHAMPAIGN-, 2007. **11**(3): p. 276.

4. Papi, E., P.J. Rowe, and V.M. Pomeroy, *Analysis of gait within the uncontrolled manifold hypothesis: Stabilisation of the centre of mass during gait.* Journal of biomechanics, 2015. **48**(2): p. 324-331.

5. Scholz, J.P. and G. Schöner, *The uncontrolled manifold concept: identifying control variables for a functional task.* Experimental brain research, 1999. **126**(3): p. 289-306.

6. Schöner, G. and J.P. Scholz, *Analyzing Variance in Multi-Degree-of-Freedom Movements: Uncovering Structure Versus Extracting Correlations.* Motor Control, 2007. **11**(3): p. 259-275.

7. Todorov, E., *Optimality principles in sensorimotor control.* Nature neuroscience, 2004. **7**(9): p. 907-915.

8. Ivanenko, Y.P., et al., *Modular control of limb movements during human locomotion.* J Neurosci, 2007. **27**(41): p. 11149-61.

9. Cusumano, J.P. and P. Cesari, *Body-goal variability mapping in an aiming task.* Biological cybernetics, 2006. **94**(5): p. 367-379.

10. Dingwell, J.B., J. John, and J.P. Cusumano, *Do humans optimally exploit redundancy to control step variability in walking?* PLoS Comput Biol, 2010. **6**(7): p. e1000856.

11. Monaco, V., et al., *Uncontrolled manifold hypothesis: Organization of leg joint variance in humans while walking in a wide range of speeds.* Human movement science, 2018. **57**: p. 227-235.

12. Müller, H. and D. Sternad, *A randomization method for the calculation of covariation in multiple nonlinear relations: illustrated with the example of goal-directed movements.* Biological cybernetics, 2003. **89**(1): p. 22-33.

13. Sternad, D., *It's not (only) the mean that matters: variability, noise and exploration in skill learning.* Current opinion in behavioral sciences, 2018. **20**: p. 183-195.
